# The invariant theory of chiral order parameters

**DOI:** 10.1063/4.0000968

**Published:** 2025-10-27

**Authors:** Carter R. Baldwin, Isaac R. Burkholder, Jeremy B. Ruebush, Harold T. Stokes, Branton J. Campbell

**Affiliations:** 1 Brigham Young University, Provo, Utah, USA; 2 Brigham Young University Idaho, Rexburg, Idaho, USA

## Abstract

In the Landau theory of phase transitions, the free energy of a system is expanded as a sum over the ring of invariant polynomials in the components of order parameters (e.g. atomic displacements, magnetic moments, lattice strains, polyhedral rotations, order-disorder parameters, etc.) belonging to irreducible representation of the parent symmetry group. The physical characteristics of the phase transition can then be understood in terms of the non-zero coefficients of the expansion. Features of Landau theory can also be applied to understand the properties of the low-symmetry phase when the parent phase employed is not physically accessible. In the present work, we explore the invariant polynomials that arise in phase transitions and otherwise-distorted crystal structures involving chiral order parameters, which can then be exploited to clearly define chiral structural features and their contributions to the free energy.

